# Peripheral White Blood Cell Subsets in Metastatic Colorectal Cancer Patients Treated with Cetuximab: The Potential Clinical Relevance

**DOI:** 10.3389/fimmu.2017.01886

**Published:** 2018-01-04

**Authors:** Ivana Z. Matić, Branka Kolundžija, Ana Damjanović, Jelena Spasić, Davorin Radosavljević, Marija Đorđić Crnogorac, Nađa Grozdanić, Zorica D. Juranić

**Affiliations:** ^1^Institute of Oncology and Radiology of Serbia, Belgrade, Serbia

**Keywords:** metastatic colorectal cancer, cetuximab, CD16, CD56, epidermal growth factor receptor IgG autoantibody

## Abstract

It was demonstrated that cetuximab-induced tumor regression is based on the effects exerted by immune cells included mainly in the innate immune response. Therefore, the focus of this study was to explore the alterations in the percentages of CD16+, and/or CD56+ lymphocytes, which are comprised of NK cells, and minority of CD56+CD3+ cells, in patients with metastatic colorectal cancer before or 2 months after the treatment with cetuximab-based regimens associated with the response to therapy. The changes in the percentages of lymphocytes and granulocytes in these patients were evaluated as well. We enrolled 50 patients with *wild-type KRAS* metastatic colorectal cancer. Disease progression was observed in 11/50 patients (non-responders), while other patients achieved partial response or stable disease (responders). Control groups included up to 72 healthy individuals. A significant decrease in the percentages of CD56+ and CD16+CD56+ lymphocytes together with a significant decrease in the percentage of lymphocytes and an increase in the ratio of granulocyte to lymphocyte percentages were observed in patients with metastatic colorectal cancer before therapy, compared with those in the healthy individuals. In contrast to those in the responders, the percentage of CD16+ lymphocytes in the overall white blood cell pool was shown to be significantly decreased in the non-responders, together with a significantly decreased percentage of lymphocytes, a significantly increased percentage of granulocytes, and an increased ratio of granulocyte to lymphocyte percentages before treatment compared with those in the healthy controls. Two months after the initiation of the treatment, significantly decreased percentages of CD16+, CD56+, and CD16+CD56+ lymphocytes were observed in patients, compared with those determined in the healthy controls. The same changes in the amounts of circulating immune cells were also observed in the responder subgroup, but the percentages of CD16+, CD56+, and CD16+CD56+ lymphocytes 2 months after treatment in the non-responder group did not differ significantly in comparison with healthy individuals. Considerable alterations of immune cell percentages observed in patients with metastatic colorectal cancer with disease progression indicate that the assessment of peripheral white blood cell architecture before treatment initiation may be clinically relevant.

## Introduction

Cetuximab (Erbitux^®^, Merck KGaA, Darmstadt, Germany) is a recombinant human–mouse chimeric monoclonal IgG1 antibody, directed against the extracellular domain of the epidermal growth factor receptor (EGFR) (1–4). This EGFR-targeting monoclonal antibody has been used as a single agent or in combination with chemotherapeutics for the treatment of patients with *wild-type KRAS* and EGFR-expressing metastatic colorectal cancer (1–4). Many clinical research studies demonstrated the efficacy of cetuximab and showed that it improves the median overall survival, progression-free survival, and response rate in these patients (3–6).

Cetuximab anticancer effects are based on the direct inhibition of EGFR activation and downstream signaling, antibody-dependent cell-mediated cytotoxicity (ADCC), complement-dependent cytotoxicity (CDC), and adaptive immune response mediated by CD8+ cytotoxic T lymphocytes (2, 7, 8). Cetuximab specifically binds to the EGF receptor and prevents the binding of its ligands, EGF and TGF-α, and inhibits the activation of EGFR/PI3K/AKT/mTOR, EGFR/RAS/RAF/MAPK/ERK, and JAK/STAT signaling pathways in cancer cells, which further leads to G1 cell cycle arrest and the induction of apoptosis, as well as a decrease in the production of matrix metalloproteinases and vascular endothelial growth factor (2, 7, 8). The ADCC, mediated by the immune system cells expressing Fcγ receptors (CD16, CD32, and CD64), plays an important role in the efficacy of tumor-antigen-specific IgG1 monoclonal antibody therapeutics, including cetuximab (7–9). CD16+ and/or CD56+ cells are considered the main effectors of the ADCC triggered by the cetuximab against cancer cells overexpressing EGFR (10, 11). The Fc fragment of cetuximab can also bind to the C1q and activate the classical complement pathway which results in cancer cell killing (9).

Considering the indispensable role of the immune-mediated mechanisms underlying the anticancer activity of cetuximab, the profiling of immune system cell subsets in the peripheral blood of patients with metastatic colorectal cancer may contribute to the characterization of individual anticancer immune response and its effects on the clinical outcome. Therefore, the aim of this study was to explore the alterations in the percentages of CD16+, and/or CD56+ lymphocytes, which are comprised of NK cells, and minority of CD56+ CD3+ cells, in patients with metastatic colorectal cancer before or 2 months after the treatment with cetuximab-based regimens associated with the response to therapy. The changes in the percentages of lymphocytes and granulocytes in these patients were evaluated as well.

## Patients and Methods

### Patients

This study included 50 patients with the *wild-type KRAS* metastatic cancer of the colon or rectum. *BRAF* mutational status of these patients was unknown. Patients were treated with cetuximab in the third line setting. Cetuximab was administered in the dose of 500 mg/m^2^ in combination with irinotecan in the dose of 180 mg/m^2^ in 2-week intervals, or as monotherapy in the same regimen. Blood samples were collected prior to the initiation of cetuximab treatment and 2 months after the beginning of the therapy, when the response was evaluated according to Response Evaluation Criteria in Solid Tumors version 1.1 (12).

The control group consisted of up to 72 healthy individuals. This study was approved and carried out in accordance with the recommendations of Ethics Committee of the Institute of Oncology and Radiology of Serbia with written informed consent from all subjects. All subjects gave written informed consent in accordance with the Declaration of Helsinki. Patients were enrolled between February 2013 and December 2015.

### Flow Cytometry Analysis

**Figure 1:**
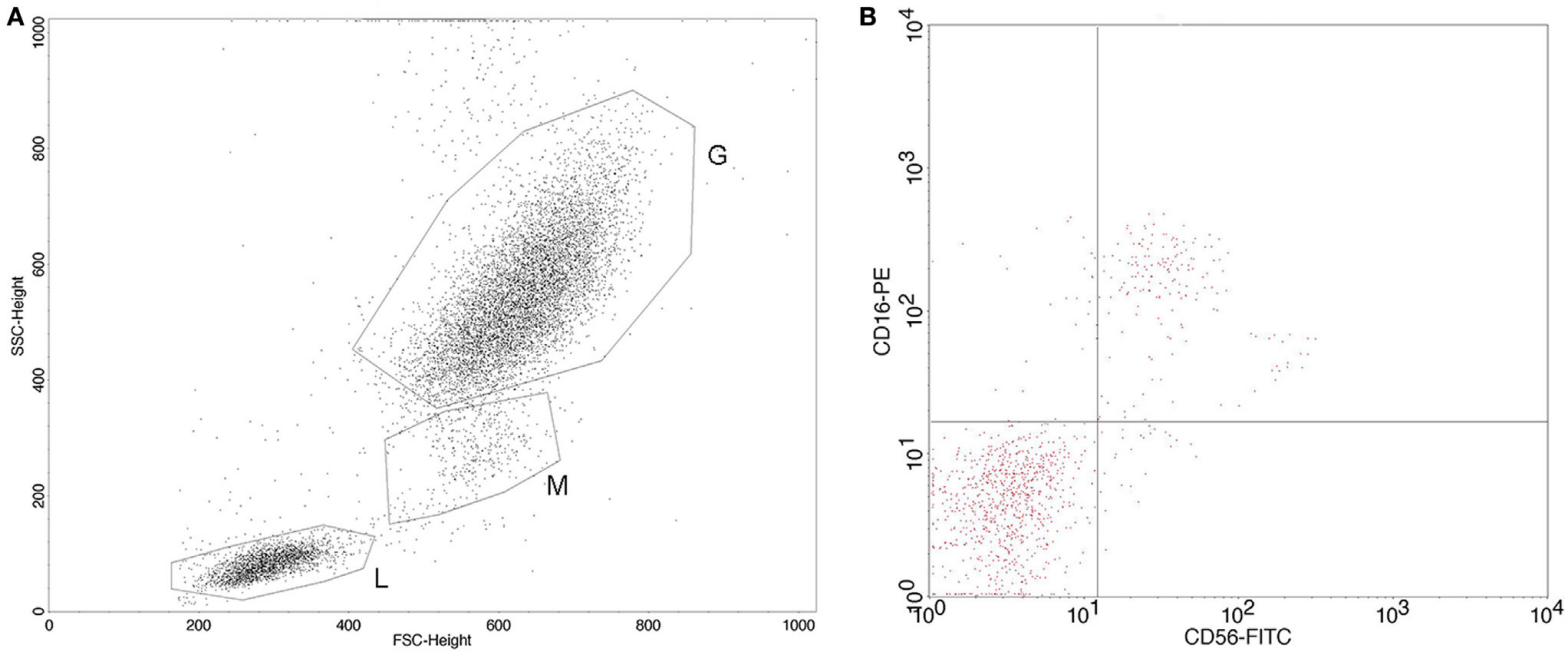
**(A)** Representative flow cytometry dot plot of white blood cell subpopulations physical gating (L, lymphocytes; M, monocytes; G, granulocytes); **(B)** representative plot of CD56–FITC vs CD16–PE staining with gate set around the lymphocytes.

Identification of specific white blood cell subpopulations—lymphocytes, granulocytes, CD16+ lymphocytes, CD56+ lymphocytes, and CD16+CD56+ lymphocytes in whole blood samples—was performed by flow cytometry. Monoclonal antibody specific for CD56 was FITC-stained, isotype mouse BALB/c IgG2b, κ, clone NCAM16.2, while monoclonal antibody specific for CD16 was PE-stained, isotype mouse BALB/c IgG1, κ, clone B73.1 (Becton Dickinson Immunocytometry Systems, CA, USA). The percentages of circulating immune system cells were determined using a FACSCalibur flow cytometer (BD Biosciences, Franklin Lakes, NJ, USA). CELLQuest software (BD Biosciences) was used for the analysis of the acquired data. The flow cytometry data are shown as the percentage of CD16 and/or CD56 expression on lymphocytes sorted by physical gating, and also on the total white blood cell pool. The percentages of lymphocytes and granulocytes were determined by white blood cells physical gating using FSC vs SSC dot plot. The representative dot plot and plot of CD56–FITC and CD16–PE staining showing CD56+, CD16+, and CD16+CD56+ lymphocytes are shown in Figure 1. The reference cut-off values (mean value and SD, Xav ± SD) for the investigated parameters were previously established in our laboratory by analyzing the blood samples of 41 healthy individuals (13).

### Determination of Anti-EGFR Antibody Concentration

The concentrations of anti-EGFR IgG autoantibodies in the sera of patients and healthy controls were determined by ELISA using recombinant human EGFR (Sino Biological Inc., Beijing, P.R. China; catalog number 10001-H08H). Briefly, 100 µL of EGFR solution in bicarbonate buffer (pH 9.5) was added to polystyrene 96-well microtiter ELISA plates at a concentration of 2.5 µg/mL (F96 MaxiSorp Nunc Immuno Plates, Thermo Fisher Scientific, Kamstrupvej, Roskilde, Denmark). The plates with coated wells were incubated at 4°C for 24 h, in order for antigens to bind to the polystyrene surface. The following day these plates were washed and polystyrene surface was blocked with 1% bovine serum albumin (BSA; Sigma, Saint Louis, MO, USA) for 1 h at room temperature. After washing and aspiration, 50 µL of serum samples (1/100) was added to wells and incubated for 1 h at room temperature, and they were subsequently aspirated and washed. Sheep anti-human IgG horseradish peroxidase-labeled secondary antibodies were used for the detection of primary antibodies bound to EGFR antigen (AP004; Binding Site, Birmingham, UK). Secondary antibody solution (50 µL) was added to the wells, plates were incubated for 1 h at room temperature, and washed five times. A substrate solution, TMB (3,3′,5,5′-tetramethylbenzidine) (INEP, Zemun, Serbia) was added to the wells (100 µL) and following a short incubation (5–10 min), the enzyme reaction was terminated by the addition of 2 M sulfuric acid (50 µL). Optical density (OD) was measured at 450 nm using microplate reader (Multiskan EX; Thermo Scientific, Waltham, MA, USA).

Cetuximab was used for the construction of calibration curves, and anti-EGFR IgG antibody concentrations were presented as ng/mL. The absorbance of the proper serum blank (two wells non-EGFR coated) was always subtracted from the absorbance of the corresponding serum sample (two wells coated with EGFR antigen). The reference cut-off values (Xav+2SD) were obtained by analyzing the sera of 36 apparently healthy individuals. Four healthy controls had 0 values for anti-EGFR IgG levels.

### Statistical Analyses

Statistical significance of the obtained data was estimated using the Mann–Whitney *U*-test and Wilcoxon signed rank test. Normal distribution of parameters was analyzed using the Kolmogorov–Smirnov test and Shapiro–Wilk test. *p* values <0.05 were considered statistically significant. In the case of multiple comparisons using the same data set, Bonferroni correction set the significance threshold value at 0.05/3 = 0.0167.

## Results

### Response to Therapy

Assessment of the response to cetuximab-containing therapy 2 months after its initiation showed the progression of disease (PD) in 11/50 patients with metastatic colorectal cancer. Other patients included in this study achieved partial response (26) or stable disease (12). One patient had allergic reaction after first therapy cycle and was excluded from further analyses. Patients with stable disease and partial response were considered responders, while patients with PD were considered non-responders.

### Peripheral White Blood Cell Subset Architecture in Patients with Metastatic Colorectal Cancer before Therapy

A significant decrease in the percentage of CD56+ and CD16+CD56+ lymphocytes was determined in the patients with metastatic colorectal cancer before the initiation of cetuximab-based therapy, when compared with healthy control individuals (Table 1). In addition, significantly decreased percentages of CD16+, CD56+, and CD16+CD56+ lymphocytes in the overall white blood cell pool were observed in the patient group before therapy, compared with those in the healthy controls. Significantly lower percentage of total lymphocytes and a significantly higher ratio of granulocyte to lymphocyte percentages were observed in patients with metastatic colorectal cancer in comparison with these values obtained for the healthy individuals.

**Table 1:** White blood cell subsets in patients with metastatic colorectal cancer before treatment with cetuximab-based regimens and in healthy controls.

	Patients with mCRC	Patients with mCRC (responders)	Patients with mCRC (non-responders)	Healthy controls (13)
*n*	39	29	9	41
% CD16+ lymphocytes	16.76 ± 8.78	17.39 ± 9.32	14.68 ± 7.47	19.08 ± 7.20
% CD16+ lymphocytes in the overall white blood cells	2.34 ± 1.76*	2.56 ± 1.95	1.61 ± 0.70**	2.92 ± 1.45
% CD56+ lymphocytes	5.97 ± 6.05*	6.37 ± 6.47**	4.86 ± 5.02**	15.00 ± 8.58
% CD56+ lymphocytes in the overall white blood cells	0.71 ± 0.83*	0.78 ± 0.93**	0.51 ± 0.46**	2.53 ± 2.28
*n*	42	31	10	41
% CD16+CD56+ lymphocytes	4.49 ± 4.70*	4.52 ± 4.94**	4.47 ± 4.35**	10.87 ± 6.44
% CD16+CD56+ lymphocytes in the overall white blood cells	0.62 ± 0.97*	0.70 ± 1.10**	0.38 ± 0.37**	1.81 ± 1.47
*n*	49	37	11	41
% lymphocytes	15.08 ± 8.52*	16.41 ± 9.03	10.64 ± 5.15**	20.39 ± 8.53
% granulocytes	72.32 ± 12.03	70.45 ± 12.80	79.07 ± 6.31**	68.90 ± 10.63
% granulocytes/% lymphocytes	7.56 ± 7.72*	6.46 ± 5.29	11.51 ± 12.76**	4.20 ± 2.23

*The numbers represent mean ± SD*.

*p < 0.05 when compared with healthy controls (Mann–Whitney *U*-test).

**p < 0.0167 when compared with healthy controls (Mann–Whitney *U*-test).

A significant decrease in the pre-treatment percentages of CD56+ and CD16+CD56+ lymphocytes, in total lymphocytes and in the overall white blood cell pool, was shown in the group of patients without PD (responders) and in the group of patients with PD (non-responders) compared with those in the healthy controls (Table 1). Unlike in the group of responders, the non-responders before treatment initiation showed a significantly lower percentage of CD16+ lymphocytes in the overall white blood cell numbers and a decreased lymphocyte percentage. In addition, non-responder group showed a significant increase in the percentage of granulocytes and a higher ratio of granulocyte to lymphocyte percentages in comparison with these values determined in the healthy individuals. No significant differences in the percentages of CD16+, CD56+, and CD16+CD56+ lymphocytes, determined in the pool of lymphocytes or overall white blood cells, as well as in the percentage of lymphocytes, granulocytes, and the ratio of granulocyte to lymphocyte percentages before receiving cetuximab-based therapy between responders and non-responders were found (Table 1). Non-responders before therapy were shown to have a decreased percentage of total lymphocytes, higher percentage of total granulocytes and a higher ratio of granulocyte to lymphocyte percentages than the responders, although these differences were not statistically significant (*p* = 0.0497, *p* = 0.0273, and *p* = 0.0469, respectively).

### Peripheral White Blood Cell Subset Architecture in Patients with Metastatic Colorectal Cancer after Therapy

Two months after the initiation of cetuximab-based therapy, a significantly decreased percentage of CD16+ lymphocytes, in addition to significantly decreased percentages of CD56+ and CD16+CD56+ lymphocytes in the pool of lymphocytes and in the overall white blood cell pool, were shown in the group of patients with metastatic colorectal cancer in comparison with those determined in healthy individuals (Table 2). The same changes in the peripheral CD16+, CD56+, and CD16+CD56+ lymphocytes were observed in the subgroup of responders, but not in the non-responder group, 2 months after therapy when compared with those in the healthy controls. No significant differences in the percentages of total lymphocytes and granulocytes and in the ratio of granulocyte to lymphocyte percentages were observed between patients and healthy controls and between the responders and healthy controls. In contrast to the alterations in white blood cell subsets assessed in responders, the non-responders, 2 months after the beginning of cetuximab-based therapy, had a significantly lower percentage of lymphocytes and a higher percentage of total granulocytes (*p* = 0.0176) and a significantly higher ratio of granulocyte to lymphocyte percentages than those in the healthy control group (Table 2). There were no statistically significant differences in the percentages of peripheral white blood cell subpopulations between the responders and non-responders 2 months after the start of the therapy. However, we observed that responders had lower percentage of CD16+ lymphocytes, higher percentage of lymphocytes, lower percentage of granulocytes, and lower ratio of granulocyte to lymphocyte percentages in comparison with those values determined in the non-responders (*p* = 0.0359, *p* = 0.0374, *p* = 0.0481, and *p* = 0.0544, respectively).

**Table 2:** White blood cell subsets in patients with metastatic colorectal cancer 2 months after treatment with cetuximab-based regimens and in healthy controls.

	Patients with mCRC	Patients with mCRC (responders)	Patients with mCRC (non-responders)	Healthy controls (13)
*n*	33	30	3	41
% CD16+ lymphocytes	14.84 ± 9.14*	14.08 ± 9.24**	22.44 ± 2.27	19.08 ± 7.20
% CD16+ lymphocytes in the overall white blood cells	2.76 ± 2.72	2.80 ± 2.86	2.39 ± 0.41	2.92 ± 1.45
% CD56+ lymphocytes	7.50 ± 7.26*	6.73 ± 6.42**	15.21 ± 12.16	15.00 ± 8.58
% CD56+ lymphocytes in the overall white blood cells	1.28 ± 1.54*	1.25 ± 1.57**	1.60 ± 1.38	2.53 ± 2.28
*n*	37	34	3	41
% CD16+CD56+ lymphocytes	5.58 ± 6.05*	5.06 ± 5.68**	11.40 ± 8.47	10.87 ± 6.44
% CD16+CD56+ lymphocytes in the overall white blood cells	0.89 ± 1.06*	0.86 ± 1.08**	1.18 ± 0.95	1.81 ± 1.47
*n*	39	36	3	41
% lymphocytes	18.47 ± 12.70	19.27 ± 12.90	8.87 ± 0.75**	20.39 ± 8.53
% granulocytes	68.47 ± 15.65	67.37 ± 15.80	81.71 ± 1.67	68.90 ± 10.63
% granulocytes/% lymphocytes	5.85 ± 4.29	5.57 ± 4.35	9.26 ± 0.90**	4.20 ± 2.23

*The numbers represent mean ± SD*.

*p < 0.05 when compared with healthy controls (Mann–Whitney *U*-test).

**p < 0.0167 when compared with healthy controls (Mann–Whitney *U*-test).

No statistically significant differences in the percentages of peripheral white blood cell subsets were observed between the patients with metastatic colorectal cancer before and 2 months after start of the cetuximab-containing therapy (Table 3). Furthermore, no significant changes in white blood cell subpopulations before and 2 months after therapy initiation were shown in the responder and non-responder groups.

**Table 3:** White blood cell subsets in patients with metastatic colorectal cancer before and 2 months after treatment with cetuximab-based regimens.

	Before therapy			After therapy		
	All patients	Responders	Non-responders	All patients	Responders	Non-responders
*n*	26	23	3	26	23	3
% CD16+ lymphocytes	16.25 ± 7.55	16.13 ± 7.85	17.24 ± 5.78	15.83 ± 9.69	14.97 ± 9.97	22.44 ± 2.27
% CD16+ lymphocytes in the overall white blood cells	2.43 ± 1.93	2.54 ± 2.02	1.56 ± 0.49	2.67 ± 2.84	2.71 ± 3.03	2.39 ± 0.41
% CD56+ lymphocytes	7.02 ± 6.67	6.88 ± 6.83	8.11 ± 6.36	7.60 ± 7.28	6.61 ± 6.15	15.21 ± 12.16
% CD56+ lymphocytes in the overall white blood cells	0.81 ± 0.87	0.82 ± 0.91	0.73 ± 0.50	1.00 ± 0.93	0.92 ± 0.86	1.60 ± 1.38
*n*	30	27	3	30	27	3
% CD16+CD56+ lymphocytes	4.30 ± 4.79	4.08 ± 4.83	6.23 ± 4.78	5.82 ± 6.44	5.21 ± 6.06	11.40 ± 8.47
% CD16+CD56+ lymphocytes in the overall white blood cells	0.54 ± 0.74	0.54 ± 0.78	0.56 ± 0.38	0.77 ± 0.87	0.72 ± 0.87	1.18 ± 0.95
*n*	38	35	3	38	35	3
% lymphocytes	15.57 ± 8.30	16.17 ± 8.36	8.58 ± 2.63	18.66 ± 12.81	19.50 ± 13.02	8.87 ± 0.75
% granulocytes	71.26 ± 11.66	70.52 ± 11.82	79.96 ± 4.38	68.2 ± 15.79	67.08 ± 15.93	81.71 ± 1.67
% granulocytes/% lymphocytes	6.48 ± 4.68	6.18 ± 4.70	9.95 ± 3.11	5.83 ± 4.35	5.53 ± 4.40	9.26 ± 0.90

*The numbers represent mean ± SD*.

*The statistical significance of obtained data was evaluated by Wilcoxon signed rank test. No statistically significant differences were observed*.

### Anti-EGFR IgG Antibodies in Patients with Metastatic Colorectal Cancer

**Figure 2:**
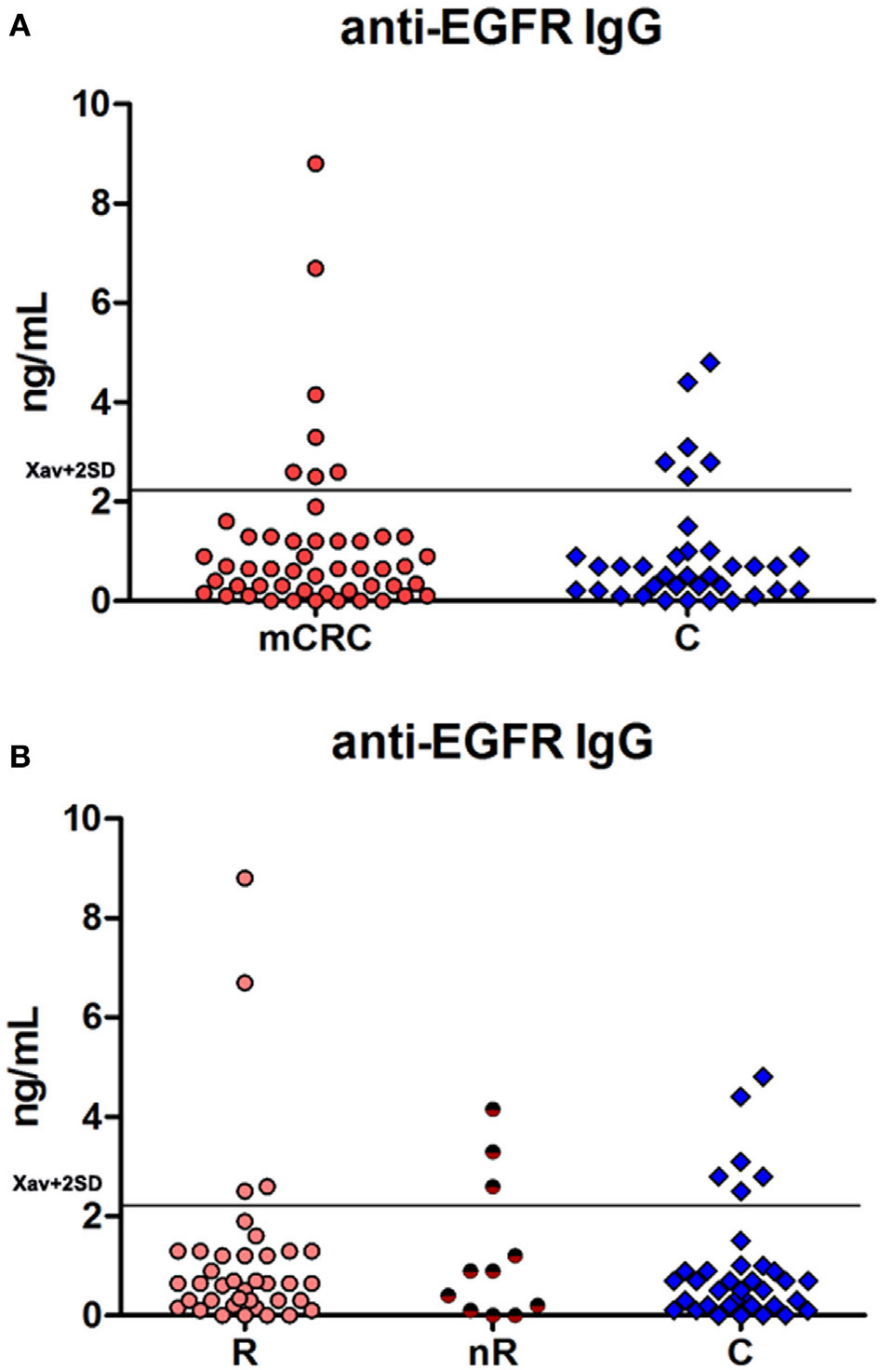
**(A)** Anti-EGFR IgG autoantibodies in patients with metastatic colorectal cancer before treatment with cetuximab-based regimens and in healthy controls; **(B)** anti-EGFR IgG autoantibodies in responders (R) and non-responders (nR) before treatment with cetuximab-based regimens and in healthy controls.

IgG antibodies specific for the extracellular domain of EGFR were detected in the sera of patients with metastatic colorectal cancer before receiving cetuximab (Figure 2). Anti-EGFR IgG autoantibodies were found in the sera of healthy individuals as well, and no significant differences in anti-EGFR IgG autoantibody levels were observed between the patients and healthy controls, and between the responders and non-responders before therapy.

## Discussion

Although ADCC can be mediated by CD16+CD56+ cells, CD16+ T lymphocytes, CD16+ monocytes and macrophages, and CD16+ granulocytes, it was suggested that CD16+CD56+ cells are the main ADCC effectors (14, 15). The focus of this investigation was on evaluation of subpopulations of CD16+ and/or CD56+ lymphocytes, which are directly involved in the cetuximab antitumor action. One of the modes of the antitumor action of cetuximab is through direct contact of this antibody bound by Fc fragment to its receptor Fcgamma R IIIA presented on CD16+ lymphocytes and by its Fab fragment to tumor cell. CD56, neural cell adhesion molecule (NCAM) is substantial for cell migration, through binding to other surface, by homotypic adhesion. Therefore, the CD16+ and/or CD56+ lymphocytes could be of importance for the migration of effector lymphocytes to sites where they could exert their antitumor action through ADCC. This mode of action is a contribution to the innate and adoptive antitumor immunity which is already present in patients, and is mediated by CD56+ lymphocytes [comprising of NK (CD56+ CD3−) and minority of NKT cells (CD56+ CD3+)]. Maréchal et al. (14) demonstrated that tumor-infiltrating CD56+ cells represent the major cetuximab-mediated ADCC effectors and that they may be valuable prognostic factors. The CD56+ T cells could also be the cells with the adoptive antitumor action-cytolytic T cells (16). In some circumstances, CD56+ T cells could differentiate into NK cells. Although CD56+ T cells have been called “NK-like,” Chan et al. (17) found that highly purified resting KIR+ CD56+ T cells were tolerant to standard NK-susceptible targets, but that after their stimulation with specific interleukins, they could kill susceptible NK target. Using gene expression analyses they showed that KIR− CD56+ T cells are metabolically active cells, also directed to effector differentiation.

Here, we demonstrated that patients with metastatic colorectal cancer before receiving cetuximab-containing therapy showed a significant decrease in the percentages of CD16+, CD56+, and CD16+CD56+ lymphocytes in the total white blood cell pool, which was accompanied by a significant decrease in lymphocyte percentages and a significant increase in the granulocyte to lymphocyte ratio, indicating tumor-specific immune alterations. Our results indicate the suppression of the immune functions in patients with metastatic colorectal cancer, and that this imbalance in the circulating immune cells may be involved in disease progression. The lower pre-treatment percentages of circulating CD56+ and CD16+CD56+ lymphocytes may not represent the limiting factor for ADCC efficiency (14).

However, study by Rocca et al. (18) demonstrated an increase in peripheral blood NK (CD3−CD56+CD16+) cell proportions among the lymphocyte subset in patients with colorectal cancer, together with considerable phenotypic and functional changes of peripheral blood NK cells in patients with colorectal cancer (18). The reason for lower percentage of CD56+ lymphocytes found in our research could be the fact that different samples of immune cells were analyzed; we analyzed immune cells from whole heparinized blood, while Rocca et al. analyzed NK cells from isolated peripheral blood mononuclear cells. In addition, CD16+ and CD56+ lymphocytes which we analyzed are comprised of several subpopulations; the major ones described being the NK cells (and their various subsets based on CD56high or CD56 dim expression), NK-like (CD56+) T cells, and invariant NKT cells (Valpha24 invariant TCR). Therefore, decrease in the percentage of CD16+ and/or CD56+ subpopulations of lymphocytes observed in CRC patients, in this work might be the consequence of more pronounced decrease in the percentages of CD56+ T cells, or decrease in the percentages of invariant NKT cells, both in comparison with percentages of NK cells.

A significant decrease in the percentages of CD56+ and CD16+CD56+ lymphocytes was detected in both responders and non-responders before therapy. However, non-responders alone were shown to have a significantly lower percentage of CD16+ lymphocytes, significantly lower percentage of total lymphocytes, significantly increased percentage of total granulocytes, and the higher ratio of granulocyte to lymphocyte percentages. Furthermore, the lower percentage of lymphocytes and the higher percentage of granulocytes and higher ratio of granulocyte to lymphocyte percentages were observed in non-responders before therapy initiation, compared with those in the responder group. Taken together, these results indicate considerable alterations in the pre-treatment peripheral white blood cell subset architecture in the non-responder subgroup. Therefore, the evaluation of these circulating immune system cells, especially CD16+ lymphocytes, in patients with metastatic colorectal cancer before treatment initiation may be useful for predicting the response to treatment with cetuximab. This is in accordance with the previously published data that showed the potential predictive significance of cetuximab-induced ADCC activity exerted by CD56+CD3− NK cells isolated from peripheral blood of patients with metastatic colorectal cancer (11). Moreover, in a previous study, it was demonstrated that cetuximab-induced tumor regression is based on the effects exerted by immune cells included in the innate and adaptive immune response, including NK cells, CD8+ T lymphocytes, and dendritic cells (19), which supports the importance of evaluating immune responses triggered by cetuximab. It should be noted that the lower number of patients in the non-responder subgroup represents a limitation for the statistical analyses of differences between responders and non-responders in this study.

Two months after receiving cetuximab, percentages of CD16+, CD56+, and CD16+CD56+ lymphocytes remained significantly decreased in the group of patients with metastatic colorectal cancer compared with those in the healthy individuals. In contrast to the differences observed before the initiation of the therapy, lymphocyte percentage and the ratio of granulocyte to lymphocyte percentages in patients were comparable to these values determined in the healthy control group. The same changes in the amounts of circulating immune cells showed in the whole group of patients were also observed in the responder subgroup. This effect could be connected with cytokine activation and immune cell stimulation which led to marked decrease in CD16 expression as Romee et al. (20) showed that activation of CD56dim NK cells by cross-linking CD16 with monoclonal antibodies resulted in a loss of CD16 (and CD62L), which correlated with increased interferon-γ production.

However, the percentages of CD16+, CD56+ and CD16+CD56+ lymphocytes 2 months after treatment in the non-responder group did not differ significantly in comparison with healthy individuals. This finding might point to the absence of immune cell activation after cross-linking of CD16 with monoclonal antibodies (20), but at least partially, might indicate that migrational capability of these, by cetuximab primed effector cells, from periphery to the tumor site failed in this group of patients. Importantly, the non-responders had lower percentage of lymphocytes, higher percentage of granulocytes, and higher ratio of granulocyte to lymphocyte percentages compared with those in the healthy controls and the responders. Decreased percentage of lymphocytes and increased ratio of granulocyte to lymphocyte percentages observed only in the non-responder subgroup before and 2 months after receiving cetuximab suggest that these changes may be implicated in cancer progression and may affect the clinical outcome. These results are in line with a meta-analysis showing that the increased preoperative neutrophil to lymphocyte ratio is associated with poor clinical outcome (21).

It has been suggested that the exploration of cellular and humoral immune response specific for EGFR might be useful in elucidation of the significance of the anticancer immune response in EGFR-overexpressing tumors (22). Furthermore, the stronger humoral immune response against EGFR may be associated with better clinical outcome (22). Pandey et al. (23) reported that higher plasma anti-EGFR IgG antibody levels are associated with the increased survival of glioblastoma patients. Anti-EGFR autoantibody levels were also determined in the sera of breast cancer patients as well (24) and showed that they were negatively correlated with the disease-free survival only in the group of breast cancer patients, with relapse or death. The humoral IgG immune response against EGFR was investigated in patients with non-small cell lung cancer receiving gefitinib and it was suggested that determining anti-EGFR IgG antibody levels may have prognostic significance (25). We did not determine any differences in the anti-EGFR IgG autoantibody levels between responders and non-responders, and patients and healthy controls. These results are in agreement with previous study in which no significant differences in EGFR autoantibodies were found between breast cancer patients and healthy individuals (24).

## Conclusion

In conclusion, our study showed significantly decreased percentages of CD16+, CD56+, and CD16+CD56+ lymphocytes in the peripheral blood of patients with metastatic colorectal cancer before therapy and after 2 months of receiving cetuximab. Significantly decreased percentage of lymphocytes and significantly increased ratio of granulocyte to lymphocyte percentages observed only in the subgroup of non-responders before and 2 months after treatment initiation when compared with healthy individuals indicate more pronounced imbalance in the immune system cells in patients who did not achieve therapy response. The finding that the percentages of CD16+, CD56+, and CD16+CD56+ lymphocytes 2 months after treatment, only in the non-responder subgroup did not differ significantly in comparison with healthy individuals opens the question whether, at least partially, the cytokine activation and immune cell stimulation of these, by cetuximab primed effector cells, failed in this subgroup of patients.

## Ethics Statement

This study was approved and carried out in accordance with the recommendations of Ethics Committee of the Institute of Oncology and Radiology of Serbia with written informed consent from all subjects. All subjects gave written informed consent in accordance with the Declaration of Helsinki.

## Author Contributions

IM performed experiments, analyzed and interpreted obtained data, and wrote the first and last version of the manuscript. BK performed experiments, interpreted obtained data, participated in writing the manuscript, and revised it critically for important intellectual content. AD, MĐC, and NG performed experiments, interpreted obtained data, and participated in writing the manuscript. JS and DR enrolled patients in the study, interpreted obtained data, and critically revised the manuscript for important intellectual content. ZJ designed the study, interpreted data, participated in writing the manuscript, and critically revised the manuscript for important intellectual content. All authors have read and approved the final version of the manuscript. All authors have agreed to be accountable for all aspects of the work in ensuring that questions related to the accuracy or integrity of any part of the work are appropriately investigated and resolved.

## Conflict of Interest Statement

The authors report no conflicts of interest. The research was conducted in the absence of any commercial or financial relationships that could be construed as a potential conflict of interest.

## References

[1] Galizia G Lieto E De Vita F Orditura M Castellano P Troiani T Cetuximab, a chimeric human mouse anti-epidermal growth factor receptor monoclonal antibody, in the treatment of human colorectal cancer. Oncogene (2007) 26:3654–60. 10.1038/sj.onc.1210381 17530019

[2] Vincenzi B Zoccoli A Pantano F Venditti O Galluzzo S. Cetuximab: from bench to bedside. Curr Cancer Drug Targets (2010) 10:80–95. 10.2174/156800910790980241 20088790

[3] Van Cutsem E Cervantes A Adam R Sobrero A Van Krieken JH Aderka D ESMO consensus guidelines for the management of patients with metastatic colorectal cancer. Ann Oncol (2016) 27:1386–422. 10.1093/annonc/mdw235 27380959

[4] García-Foncillas J Díaz-Rubio E. Progress in metastatic colorectal cancer: growing role of cetuximab to optimize clinical outcome. Clin Transl Oncol (2010) 12:533–42. 10.1007/s12094-010-0551-3 20709651

[5] Van Cutsem E Köhne CH Hitre E Zaluski J Chang Chien CR Makhson A Cetuximab and chemotherapy as initial treatment for metastatic colorectal cancer. N Engl J Med (2009) 360:1408–17. 10.1056/NEJMoa0805019 19339720

[6] Sorich MJ Wiese MD Rowland A Kichenadasse G McKinnon RA Karapetis CS. Extended RAS mutations and anti-EGFR monoclonal antibody survival benefit in metastatic colorectal cancer: a meta-analysis of randomized, controlled trials. Ann Oncol (2015) 26:13–21. 10.1093/annonc/mdu378 25115304

[7] Trivedi S Concha-Benavente F Srivastava RM Jie HB Gibson SP Schmitt NC Immune biomarkers of anti-EGFR monoclonal antibody therapy. Ann Oncol (2015) 26:40–7. 10.1093/annonc/mdu156 24997207 PMC4269339

[8] Holubec L Polivka J Jr Safanda M Karas M Liska V. The role of cetuximab in the induction of anticancer immune response in colorectal cancer treatment. Anticancer Res (2016) 36:4421–6. 10.21873/anticanres.10985 27630277

[9] Zhuang H Xue Z Wang L Li X Zhang N Zhang R Efficacy and immune mechanisms of cetuximab for the treatment of metastatic colorectal cancer. Clin Oncol Cancer Res (2011) 8:207–14. 10.1007/s11805-011-0582-8

[10] Lee SC Srivastava RM López-Albaitero A Ferrone S Ferris RL. Natural killer (NK): dendritic cell (DC) cross talk induced by therapeutic monoclonal antibody triggers tumor antigen-specific T cell immunity. Immunol Res (2011) 50:248–54. 10.1007/s12026-011-8231-0 21717064 PMC3415245

[11] Monteverde M Milano G Strola G Maffi M Lattanzio L Vivenza D The relevance of ADCC for EGFR targeting: a review of the literature and a clinically-applicable method of assessment in patients. Crit Rev Oncol Hematol (2015) 95:179–90. 10.1016/j.critrevonc.2015.02.014 25819749

[12] Eisenhauer EA Therasse P Bogaerts J Schwartz LH Sargent D Ford R New response evaluation criteria in solid tumours: revised RECIST guideline (version 1.1). Eur J Cancer (2009) 45:228–47. 10.1016/j.ejca.2008.10.026 19097774

[13] Rašković S Matić IZ Ðordić M Damjanović A Kolundžija B Grozdanić-Stanisavljević N Immunoreactivity to food antigens in patients with chronic urticaria. Immunol Invest (2014) 43:504–16. 10.3109/08820139.2014.892509 24661189

[14] Maréchal R De Schutter J Nagy N Demetter P Lemmers A Devière J Putative contribution of CD56 positive cells in cetuximab treatment efficacy in first-line metastatic colorectal cancer patients. BMC Cancer (2010) 10:340. 10.1186/1471-2407-10-340 20591136 PMC2912265

[15] Veluchamy JP Spanholtz J Tordoir M Thijssen VL Heideman DA Verheul HM Combination of NK cells and cetuximab to enhance anti-tumor responses in RAS mutant metastatic colorectal cancer. PLoS One (2016) 11:e0157830. 10.1371/journal.pone.0157830 27314237 PMC4912059

[16] Caligiuri MA. Human natural killer cells. Blood (2008) 112:461–9. 10.1182/blood-2007-09-077438 18650461 PMC2481557

[17] Chan WK Rujkijyanont P Neale G Yang J Bari R Das Gupta N Multiplex and genome-wide analyses reveal distinctive properties of KIR+ and CD56+ T cells in human blood. J Immunol (2013) 191:1625–36. 10.4049/jimmunol.1300111 23858032 PMC4275795

[18] Rocca YS Roberti MP Juliá EP Pampena MB Bruno L Rivero S Phenotypic and functional dysregulated blood NK cells in colorectal cancer patients can be activated by cetuximab plus IL-2 or IL-15. Front Immunol (2016) 7:413 10.3389/fimmu.2016.00413 27777574 PMC5056190

[19] Yang X Zhang X Mortenson ED Radkevich-Brown O Wang Y Fu YX. Cetuximab-mediated tumor regression depends on innate and adaptive immune responses. Mol Ther (2013) 21:91–100. 10.1038/mt.2012.184 22990672 PMC3538305

[20] Romee R Foley B Lenvik T Wang Y Zhang B Ankarlo D NK cell CD16 surface expression and function is regulated by a disintegrin and metalloprotease-17 (ADAM17). Blood (2013) 121:3599–608. 10.1182/blood-2012-04-425397 23487023 PMC3643761

[21] Malietzis G Giacometti M Kennedy RH Athanasiou T Aziz O Jenkins JT. The emerging role of neutrophil to lymphocyte ratio in determining colorectal cancer treatment outcomes: a systematic review and meta-analysis. Ann Surg Oncol (2014) 21:3938–46. 10.1245/s10434-014-3815-2 24866438

[22] Sasada T Azuma K Ohtake J Fujimoto Y. Immune responses to epidermal growth factor receptor (EGFR) and their application for cancer treatment. Front Pharmacol (2016) 7:405. 10.3389/fphar.2016.00405 27833557 PMC5080289

[23] Pandey JP Kistner-Griffin E Radwan FF Kaur N Namboodiri AM Black L Endogenous antibody responsiveness to epidermal growth factor receptor is associated with immunoglobulin allotypes and overall survival of patients with glioblastoma. Neuro Oncol (2015) 17:678–84. 10.1093/neuonc/nou298 25326496 PMC4482853

[24] Olsen DA Jakobsen EH Brandslund I. Quantification of EGFR autoantibodies in the amplification phenomenon of HER2 in breast cancer. Clin Chem Lab Med (2013) 51:2325–9. 10.1515/cclm-2013-0166 24021599

[25] Azuma K Komatsu N Hattori S Matsueda S Kawahara A Sasada T Humoral immune responses to EGFR-derived peptides predict progression-free and overall survival of non-small cell lung cancer patients receiving gefitinib. PLoS One (2014) 9:e86667. 10.1371/journal.pone.0086667 24497964 PMC3909003

